# HOXA5 as a Dual Modulator of Tumor Biology in Endometrial Cancer

**DOI:** 10.3390/cancers17152473

**Published:** 2025-07-26

**Authors:** Yi-Kai Fu, Ching-Yu Shih, Chiao-Yin Cheng, Hua Ho, Yen-Lin Chen

**Affiliations:** 1 Department of Emergency Medicine, Far Eastern Memorial Hospital, New Taipei 220, Taiwan; yikaifu@gmail.com (Y.-K.F.); chiaoyin810406@gmail.com (C.-Y.C.); 2 Center for Precision Medicine and Genomics, Tri-Service General Hospital, National Defense Medical University, Taipei 114, Taiwan; c588011124@gmail.com; 3 Department of Pathology, Center for Precision Medicine and Genomic, Tri-Service General Hospital, National Defense Medical University, Taipei 114, Taiwan

**Keywords:** endometrial cancer, HOXA5, fibronectin, ki-67, CD31

## Abstract

**Background/Objectives**: Endometrial cancer (EC) is the most prevalent gynecological malignancy, with increasing incidence and mortality. HOXA5, a developmental transcription factor, has been linked to prognosis in various cancers, but its role in EC remains unclear. This study aimed to evaluate the prognostic potential of HOXA5 in EC and to explore its association with common tumor-related proteins. **Methods**: We analyzed 75 EC tissue samples using immunohistochemistry to evaluate HOXA5 expression and its association with clinicopathological features and tumor-related biomarkers, including Ki-67, CD31, and fibronectin. Statistical analyses included logistic regression and Kaplan–Meier survival analysis. **Results**: High HOXA5 expression was significantly associated with elevated Ki-67 levels (*p* = 0.001) but paradoxically correlated with improved overall survival (*p* = 0.026). CD31 and fibronectin levels were significantly lower in the high-HOXA5 group (*p* = 0.007 and *p* = 0.001, respectively), suggesting reduced angiogenic and invasive potential. However, neither marker remained significant in multivariable analysis. **Conclusions**: HOXA5 may exert a dual role in EC by promoting proliferation while limiting tumor progression via suppression of angiogenesis and matrix remodeling. It holds potential as a prognostic biomarker and therapeutic target.

## Simple Summary

Endometrial cancer is the most common gynecological cancer in developed countries, but predicting how it will progress remains challenging. This study focused on a gene called *HOXA5*, which helps control cell growth and development. We examined tissue samples from 75 patients with endometrial cancer and found that high HOXA5 expression was linked to increased tumor cell growth, but at the same time it was associated with better overall survival. Interestingly, patients with high HOXA5 levels had lower amounts of markers related to blood vessel formation and tissue invasion, which may slow down cancer’s spread. Our findings suggest that HOXA5 may act like a double-edged sword—encouraging cancer cell growth while limiting their ability to spread. This means HOXA5 could be used as a helpful marker to predict patient outcomes and might even be a future target for therapy.

## 1. Introduction

Endometrial cancer (EC) is the most common gynecological malignancy in developed countries, and its incidence and mortality have been steadily rising in recent decades. This trend is largely driven by increasing rates of obesity, aging populations, and the prevalence of metabolic disorders such as diabetes and polycystic ovary syndrome. Recent global data highlight a growing burden of EC, especially in high-income countries, and indicate a shift toward younger age at diagnosis. These epidemiological trends underscore the need for enhanced prevention strategies and early detection efforts, particularly in populations with rising metabolic health challenges [1,2].

EC is associated with a variety of modifiable and non-modifiable risk factors. Obesity is the most significant factor, with each 5 kg/m^2^ increase in BMI raising the risk by approximately 60%, and central adiposity (e.g., waist-to-hip ratio) further compounding the risk. Hormonal imbalances also play a key role: prolonged unopposed estrogen exposure, use of tamoxifen, early menarche, late menopause, and anovulatory conditions such as polycystic ovary syndrome (PCOS) all elevate the risk of EC. Metabolic comorbidities, including diabetes and hypertension, are independently linked to increased EC incidence. Reproductive history also influences the disease risk; nulliparity raises the risk of EC, whereas long-term use of combined oral contraceptives reduces it. Additionally, genetic predisposition, particularly Lynch syndrome, significantly increases the lifetime risk of EC. Lifestyle factors such as physical inactivity, high-fat diets, and prior pelvic radiation further contribute to disease development. Understanding these risk factors is essential for effective prevention and early detection strategies [1,3].

In addition to the aforementioned risk factors, genetic alterations and protein expression may also influence the development and prognosis of EC. Several molecular markers have been identified as important prognostic factors in EC. Mutations in TP53, commonly found in serous-type tumors, are associated with aggressive behavior and poor prognosis [4]. In contrast, POLE mutations define an ultramutated subtype with excellent outcomes and may not require adjuvant therapy [5]. Overexpression of HER2/neu (ERBB2) is also linked to poorer survival, particularly in high-grade serous carcinomas, and may guide targeted therapy using trastuzumab [6]. HIF-1α polymorphisms may influence tumor adaptation to hypoxia and are associated with both risk and prognosis [7]. Glycodelin (PAEP) and OVGP1, both secretory proteins involved in reproductive tract function, have shown associations with EC progression and patient survival [8]. Additionally, SOWAHA (ANKRD43) has emerged as a potential tumor suppressor, with reduced expression linked to worse outcomes in several cancers, including EC [3]. These molecular alterations not only provide insights into disease biology but also offer potential targets for precision therapy.

In EC, several proteins have been implicated in tumor progression and prognosis. Caspase-3, a key mediator of apoptosis, has been shown to play a role in programmed cell death, and its altered activity may influence tumor survival [9]. Ki-67, a marker of cellular proliferation, is frequently overexpressed in high-grade EC and is associated with poor prognosis [10]. CD31, an endothelial marker used to evaluate microvessel density, reflects angiogenic activity and is associated with increased tumor aggressiveness [5]. The epithelial-to-mesenchymal transition (EMT) markers E-cadherin and N-cadherin are also critical; reduced E-cadherin and increased N-cadherin expression have been correlated with enhanced invasion and metastasis in EC [11]. Fibronectin, an extracellular matrix component, promotes cell adhesion and motility and is overexpressed in aggressive tumors [12]. Activation of key signaling pathways, including pAkt (PI3K/Akt), pErk (MAPK), and pStat3 (JAK/STAT), is frequently observed in EC and is associated with increased proliferation, survival, and immune evasion [13,14]. Finally, pAMPK, a metabolic regulator, has been implicated in tumor metabolism and may influence sensitivity to therapy [15]. These molecular markers offer valuable insights into EC’s pathobiology and may serve as targets for prognosis and treatment.

HOXA5 (Homeobox A5) is a transcription factor belonging to the HOX gene family, known for its essential role in embryonic development, cellular differentiation, and tissue homeostasis. In EC, HOXA5 overexpression has been associated with poorer overall survival and is considered to be an independent prognostic factor; it is also linked to TP53 mutations and high copy-number alterations [16].

Beyond EC, HOXA5 has been studied extensively in a wide range of malignancies and is increasingly recognized as a key regulator of tumor suppression, differentiation, and prognosis [17]. In non-small-cell lung cancer (NSCLC), reduced HOXA5 expression correlates with larger tumor size, lymph node metastasis, and worse outcomes, partially through p21-mediated growth inhibition [18]. Additional studies show that HOXA5 can inhibit cell migration and metastasis, contributing to improved patient prognosis [17]. In cervical cancer, downregulation of HOXA5 is linked to myometrial invasion, lymphatic spread, and unfavorable prognosis, possibly via the AKT/p27 pathway [19]. In gastric cancer, HOXA5 expression is significantly downregulated, and this reduction is associated with larger tumor size, advanced TNM stage, and poor survival outcomes, potentially through the modulation of p21, c-Myc, and Ki67 [20]. In prostate cancer, HOXA5 has been shown to suppress cancer cells’ stemness and malignancy by regulating the SPRY2/MEK/ERK pathway [21].

Furthermore, in breast cancer, HOXA5 functions as a tumor suppressor by enhancing p53 expression and regulating progesterone receptor activity [22]. In acute myeloid leukemia (AML), aberrant HOXA5 expression is associated with treatment response and overall survival [23]. Two comprehensive reviews [17,24] further contextualize HOXA5’s broader biological functions in both development and disease. These collective findings suggest that HOXA5 plays a tumor-suppressive role across multiple cancer types and may serve as a valuable prognostic biomarker and potential therapeutic target.

Although a recent Cancer Genome Atlas-based study reported that HOXA5 overexpression is associated with poor prognosis in EC [16], the underlying mechanisms remain unclear. Furthermore, the relationships between HOXA5 and key biomarkers associated with cellular proliferation, angiogenesis, and extracellular matrix remodeling remain largely unexplored. Therefore, this study aimed to evaluate the prognostic potential of HOXA5 in EC through immunohistochemical analysis of clinical tissue samples, and to explore its association with common tumor-related proteins.

## 2. Materials and Methods

We collected EC tissue samples from patients diagnosed between 2000 and 2014, totaling 75 specimens. All of the samples were obtained from patients with newly diagnosed tumors who had not received chemotherapy or radiotherapy prior to tissue collection. In addition, we collected basic patient information, including sex, age, tumor size, histological grade, cancer stage, and survival status. This study was approved by the Institutional Review Board of Cardinal Tien Hospital on 27 June 2018, under the approval number CTH-106-2-5-042.

For each sample, a 2 mm diameter tissue core was extracted and assembled into a tissue microarray. The cores were embedded in paraffin blocks and sectioned into 0.3 µm thick slices, which were then mounted onto glass slides for subsequent immunohistochemical analysis. Prior to staining, the slides were baked at 65 °C for 1 h to enhance tissue adhesion, followed by rehydration through sequential immersion in xylene (two washes, 10 min each), absolute ethanol (5 min), graded ethanol solutions (95% and 75%, 5 min each), and a final rinse in distilled water (10 min). Immunohistochemical staining was performed using the fully automated Ventana BenchMark XT system (Ventana Medical Systems, Tucson, AZ, USA).

Following rehydration, the tissue sections were rinsed with phosphate-buffered saline and subjected to antigen retrieval using ethylenediaminetetraacetic acid buffer for 24 min, under optimal conditions for each antibody. The primary antibodies were then incubated at 37 °C for 1 h. Detection was performed using diaminobenzidine as the chromogen, followed by hematoxylin counterstaining to visualize cell nuclei. The antibody dilutions are listed in Table 1, including HOXA5, caspase-3, Ki-67, CD31, E-cadherin, N-cadherin, fibronectin, phosphorylated Akt (p-Akt), phosphorylated Erk (p-Erk), phosphorylated STAT3 (p-STAT3), and phosphorylated AMPK (p-AMPK).

**Figure 1:**
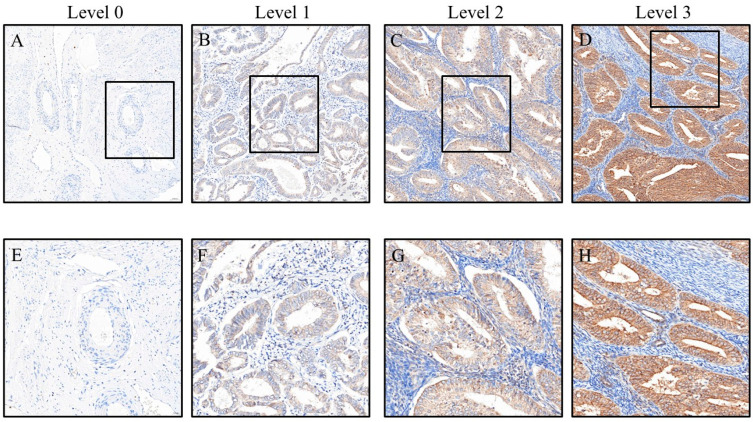
Representative images showing HOXA5 immunohistochemical staining intensity in endometrial cancer (scores 0–3). Immunohistochemical staining of EphA5 in endometrial cancer (EC) tissues, illustrating varying expression levels. Panels (**A–D**) show low- to high-power views at 200× magnification (scale bars: 50 μm), representing negative (**A**), weak (**B**), moderate (**C**), and strong (**D**) EphA5 expression, respectively. Panels (**E**–**H**) present corresponding high-magnification views at 400× (scale bars: 20 μm), highlighting the cellular localization and staining intensity of EphA5. The box indicates the magnified region. EC, endometrial cancer; EphA5, Eph receptor A5.

To ensure objective and standardized evaluation of immunostaining, all tissue slides were stained using an automated immunostaining system, operated by trained technicians who were blinded to the identity and characteristics of each specimen. Whole-slide imaging was then performed using a slide scanner (3DHISTECH, Budapest, Hungary) at 200× magnification. Regions of interest (ROIs) were identified using Pattern Quant software (Version 2.4), which was pre-trained to recognize relevant histological areas. Quantitative analysis was subsequently conducted using CellQuant (3DHISTECH), which automatically calculated the H-score by multiplying the staining intensity (0–3; 0 = no staining, 1 = faint, 2 = moderate, 3 = strong) by the percentage of positively stained area (0–100%), yielding a semi-quantitative score ranging from 0 to 300 (Figure 1). Additionally, ImageJ software (version 1.54f; NIH, Bethesda, MD, USA) was used to cross-validate the percentage of positive staining. All final results were reviewed and confirmed by Dr. Yen-Lin Chen from the Department of Pathology at Tri-Service General Hospital, who verified the automated image analysis and exported the data for further interpretation.

Samples were stratified into high- and low-HOXA5 expression groups using an H-score cutoff of 17. Continuous variable distributions were evaluated with the Kolmogorov–Smirnov test, revealing non-normal distributions for all parameters. Consequently, these variables were summarized as median values with interquartile ranges and analyzed using nonparametric Mann–Whitney U tests. Categorical data were presented as frequency counts and percentages, with between-group comparisons performed using chi-squared tests or Fisher’s exact tests for limited sample sizes. We employed a two-stage analytical approach to identify prognostic factors. First, univariate logistic regression screened for variables significantly associated with both HOXA5 expression and clinical outcomes. Subsequently, significant variables from this initial analysis were entered into multivariate logistic regression models to identify independent prognostic factors, with the results reported as adjusted odds ratios and corresponding 95% confidence intervals. Survival outcomes were compared between groups using Kaplan–Meier analysis with log-rank testing. All analyses were conducted using SPSS Statistics (version 26.0; IBM Corp., Armonk, NY, USA), with statistical significance defined as two-tailed *p*-values < 0.05.

## 3. Results

Among the 75 EC patients analyzed, the clinicopathological characteristics were compared between those with low (n = 30) and high (n = 45) HOXA5 expression, as shown in Table 2. The median age was similar between the low- and high-expression groups (56.5 [50.5–64.0] vs. 56.0 [50.0–59.5] years, *p* = 0.482). Tumor differentiation showed no significant difference between groups: well-differentiated tumors were observed in 20.0% vs. 26.7%, moderately differentiated in 56.7% vs. 57.8%, and poorly differentiated in 23.3% vs. 15.6% of the low- and high-expression groups, respectively (*p* = 0.631). Median tumor size was slightly larger in the low-expression group (3.8 cm [1.4–6.6]) compared to the high-expression group (2.8 cm [1.6–5.0]), but the difference was not significant (*p* = 0.661). Regarding cancer stage, the majority of patients in both groups were diagnosed at stage I (80.0% vs. 82.2%), followed by stage II (6.7% vs. 8.9%) and stage III EC (13.3% vs. 8.9%), with no significant association between HOXA5 expression and stage distribution (*p* = 0.796). Overall, no statistically significant differences in age, tumor grade, size, or stage were found between the two expression groups.

**Figure 2:**
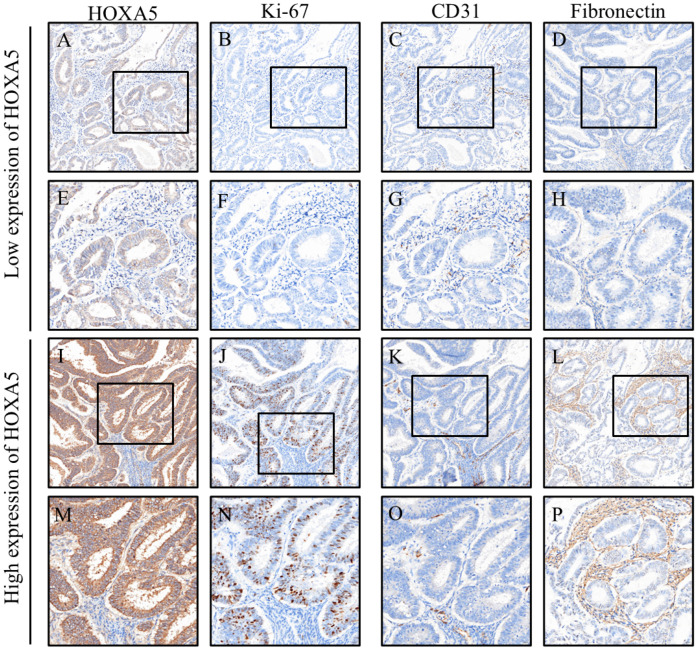
Illustrative comparison of biomarker expression patterns under low and high HOXA5 expression: (**A**–**H**) low expression of HOXA5; (**I**–**P**) high expression of HOXA5. Immunohistochemical staining of EphA5 in endometrial cancer (EC) tissues, demonstrating different expression levels. Panels (**A**–**D**) display low-magnification views (200×, scale bars: 50 μm), corresponding to negative (**A**), weak (**B**), moderate (**C**), and strong (**D**) EphA5 expression. Panels (**E**–**H**) show high-magnification images (400×, scale bars: 20 μm) of the boxed regions in (**A**–**D**), respectively, providing detailed visualization of cellular staining patterns. The box indicates the magnified region. EC, endometrial cancer; EphA5, Eph receptor A5.

Expression levels of key biomarkers were compared between the low- and high-HOXA5 expression groups, as shown in Table 3. Caspase-3 levels tended to be higher in the high-HOXA5 group (8.6 [5.4–13.7]) than in the low-expression group (6.3 [4.5–9.3]), although the difference was not statistically significant (*p* = 0.062). The proliferation marker Ki-67 showed a significantly higher expression in the high-HOXA5 group (9.0 [1.7–24.4]) compared to the low-expression group (2.3 [0.3–7.1]; *p* = 0.001), suggesting an association between HOXA5 expression and increased cellular proliferation (Figure 2).

In terms of angiogenesis, CD31 expression was significantly lower in the high-HOXA5 group (6.4 [4.8–8.4]) than in the low-expression group (8.2 [6.4–14.8]; *p* = 0.007), implying a potential inverse relationship between HOXA5 and vascular marker levels. E-cadherin expression was slightly higher in the high-expression group (110.0 [105.8–118.5]) than in the low-expression group (108.5 [101.6–115.6]), although this difference was not significant (*p* = 0.216). N-cadherin levels did not significantly differ between the groups (*p* = 0.665).

Interestingly, fibronectin expression was significantly lower in the high-HOXA5 group (2.9 [1.1–23.4]) compared to that in the low-expression group (16.6 [6.3–51.5]; *p* = 0.001), suggesting that HOXA5 may be negatively associated with extracellular matrix remodeling. For signaling molecules, *p*-Akt was significantly elevated in the high-HOXA5 group (4.1 [1.9–6.8]) compared to the low-expression group (2.5 [1.2–3.6]; *p* = 0.031), whereas *p*-Erk levels showed a non-significant trend toward higher expression in the high-HOXA5 group (*p* = 0.069). There were no significant differences in *p*-Stat3 or *p*-AMPK expression between groups (*p* = 0.414 and *p* = 0.218, respectively). These findings suggest that HOXA5 expression may be linked to increased cellular proliferation and altered signaling pathway activity, particularly involving Ki-67, fibronectin, CD31, and p-Akt.

To explore potential factors associated with high HOXA5 expression in EC, univariable and multivariable logistic regression analyses were performed (Table 4). In the univariable analysis, clinical parameters such as age (OR = 0.98, 95% CI: 0.93–1.03, *p* = 0.329), tumor size (OR = 0.94, 95% CI: 0.80–1.11, *p* = 0.450), and stage (stage II vs. I: OR = 1.30, *p* = 0.774; stage III vs. I: OR = 0.65, *p* = 0.566) were not significantly associated with HOXA5 expression. Tumor grade also showed no significant difference when comparing moderate (OR = 0.77, *p* = 0.649) or poorly differentiated tumors (OR = 0.50, *p* = 0.344) to well-differentiated ones.

Among the molecular markers, Ki-67, a well-known proliferation marker, was significantly associated with increased odds of high HOXA5 expression (OR = 1.08, 95% CI: 1.02–1.14, *p* = 0.008), suggesting that tumors with higher proliferative activity tend to have elevated HOXA5 levels. CD31, a marker of vascular endothelium and angiogenesis, showed a statistically significant inverse association (OR = 0.91, 95% CI: 0.83–0.98, *p* = 0.018), implying that reduced microvessel density may be associated with higher HOXA5 expression. Similarly, fibronectin, an extracellular matrix protein involved in adhesion and invasion, was also negatively associated (OR = 0.98, 95% CI: 0.97–1.00, *p* = 0.021), indicating that higher HOXA5 expression may be correlated with decreased fibronectin levels.

Other markers, including caspase-3 (*p* = 0.088), p-Akt (*p* = 0.597), p-Erk (*p* = 0.628), p-Stat3 (*p* = 0.163), p-AMPK (*p* = 0.257), E-cadherin (*p* = 0.189), and N-cadherin (*p* = 0.241), did not show statistically significant associations with HOXA5 expression in the univariable analysis.

In the multivariable logistic regression, markers that showed significance or borderline significance in the univariable analysis (Ki-67, CD31, and fibronectin) were included. Ki-67 remained an independent predictor of high HOXA5 expression (adjusted OR = 1.12, 95% CI: 1.03–1.19, *p* = 0.004), reinforcing its strong correlation with tumor proliferation. CD31 exhibited a borderline inverse association (adjusted OR = 0.89, 95% CI: 0.78–1.01, *p* = 0.063), while fibronectin retained a marginally significant inverse relationship (adjusted OR = 0.98, 95% CI: 0.96–1.00, *p* = 0.057). These findings suggest that high HOXA5 expression in EC may be independently associated with increased cell proliferation and decreased angiogenesis and extracellular matrix interaction.

**Figure 3:**
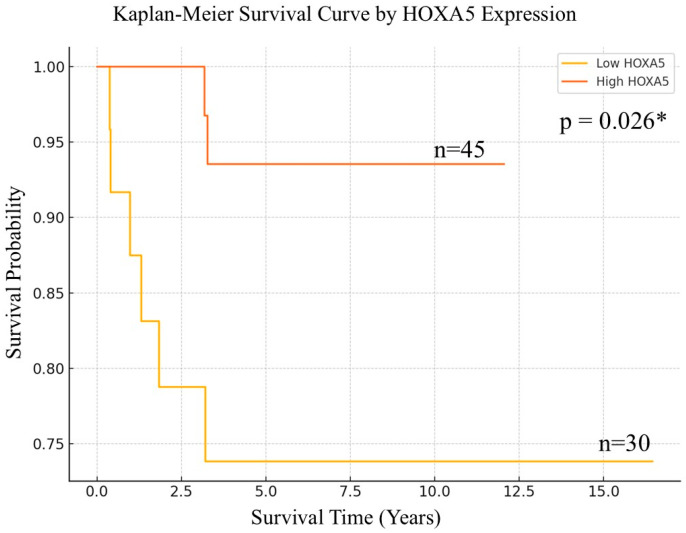
Kaplan–Meier survival curves comparing overall survival between low- and high-HOXA5 expression groups. * *p* < 0.05.

Kaplan–Meier survival analysis revealed a notable difference in overall survival between patients with low and high HOXA5 expression, using a threshold of ≤17 to define low expression. Patients in the low-HOXA5 group exhibited a steeper decline in survival probability over time, indicating a poorer prognosis, whereas those with high HOXA5 expression demonstrated more favorable survival outcomes, with a relatively stable curve throughout the follow-up period. The difference between the two groups was statistically significant, as determined by the log-rank test (*p* = 0.026), suggesting that HOXA5 may serve as a potential prognostic biomarker in EC (Figure 3).

## 4. Discussion

In summary, our study demonstrates that high HOXA5 expression is significantly associated with enhanced proliferative activity, reduced angiogenesis, and decreased extracellular matrix remodeling in EC. Immunohistochemical analysis revealed that HOXA5 expression positively correlates with Ki-67 levels while inversely correlating with CD31 and fibronectin expression. Multivariable logistic regression confirmed Ki-67 as an independent predictor of high HOXA5 expression. Furthermore, Kaplan–Meier survival analysis showed that patients with low HOXA5 expression had significantly poorer overall survival, supporting the role of HOXA5 as a prognostic biomarker. These findings suggest that HOXA5 may contribute to tumor progression by modulating key molecular pathways and hold potential as a target for prognostic stratification and future therapeutic intervention in EC. Further studies are warranted to clarify the underlying mechanisms and to explore the translational relevance of HOXA5 in clinical practice. To address concerns regarding the prognostic significance of HOXA5 in endometrial cancer (EC), we refer to the recent publication by Song et al., which analyzed data from the TCGA and GEO databases. Their study demonstrated that HOXA5 expression is significantly associated with poor prognosis in uterine corpus endometrioid adenocarcinoma (*p* = 0.044; HR = 1.832) [16]. Interestingly, this finding contrasts with the TCGA-based analysis presented in the Human Protein Atlas (https://www.proteinatlas.org/ENSG00000106004-HOXA5/cancer/endometrial+cancer, accessed on 21 July 2025), where patients with higher HOXA5 expression exhibited better overall survival [25]. This discrepancy highlights the complexity of HOXA5’s role in EC and suggests that its prognostic value may vary depending on the specific patient cohort, cutoff definition, or analytical methodology.

To address the limitation of statistical power due to our small sample size, we also conducted effect size analyses. Cohen’s d indicated moderate-to-large effects in key molecular markers, including Ki67 (d = 0.71), caspase-3 (d = 0.42), and CD31 (d = −0.64), supporting HOXA5’s potential influence on proliferation and angiogenesis. Additionally, E-cadherin (d = 0.31) and fibronectin (d = −0.60) showed small-to-moderate effects, possibly implicating HOXA5 in epithelial–mesenchymal transition (Supplementary Table S1).

In recent years, numerous studies focusing on endometrial cancer (EC) have investigated biomarkers predictive of prognosis. According to the TCGA molecular classification of EC, POLE-ultramutated tumors are typically associated with favorable outcomes, while p53-abnormal tumors are linked to poor prognosis [26,27]. At the protein level, overexpression of TP53, WFDC2, ERBB2, and L1CAM, as well as reduced expression of estrogen and progesterone receptors (ERs/PRs), have been correlated with increased tumor aggressiveness, lymphovascular invasion, and decreased overall survival in EC [28,29,30]. Notably, high L1CAM expression (>10%) has been identified as a significant prognostic marker even within the NSMP (no specific molecular profile) subtype of EC [6].

We further found that HOXA5 has been implicated in tumor suppression across various cancer types through its regulatory effects on key oncogenic and hormonal pathways. It positively regulates TP53 expression by binding to TAAT motifs in its promoter, thereby enhancing p53-mediated apoptosis and invasion suppression [31,32,33,34]. In hormone-responsive tissues such as the breast and endometrium, HOXA5 is positively correlated with progesterone receptor (PR) expression, and its loss is associated with reduced PR signaling, impaired differentiation, and altered uterine function [22,35,36,37]. Additionally, HOXA5 acts as a negative regulator of oncogenic drivers, including ERBB2 and L1CAM. Its downregulation is linked to increased tumor aggressiveness, enhanced EMT, and metastatic potential via upregulation of ERBB2 and L1CAM expression [38,39,40]. Collectively, these findings highlight HOXA5 as a multifunctional tumor suppressor influencing p53 signaling, hormonal responsiveness, and metastatic behavior.

Serum biomarkers such as CA-125 and HE4 have also been shown to correlate with advanced stage, deep myometrial invasion, and lymph node involvement in EC patients [41,42]. In addition, emerging liquid biopsy tools—such as circulating free DNA (cfDNA), circulating tumor DNA (ctDNA), and circulating tumor cells (CTCs)—have demonstrated potential in detecting early recurrence and identifying high-risk pathological features [43,44]. Furthermore, systemic inflammatory markers, including C-reactive protein (CRP) and the neutrophil-to-lymphocyte ratio (NLR), have been associated with worse cancer-specific survival in EC [45,46].

Building on these efforts to refine prognostic assessment, we propose HOXA5 as a promising novel biomarker in EC. While it is not yet as extensively validated as TP53 or L1CAM, our findings indicate that decreased HOXA5 expression is significantly associated with advanced stage and vascular invasion. Given its established tumor-suppressive role in several other malignancies, HOXA5 may serve as a valuable complementary marker to enhance molecular risk stratification in endometrial cancer. This perspective has been further elaborated in the revised Discussion section.

Multiple studies have demonstrated the prognostic and diagnostic significance of Ki-67 in EC. In early-stage patients, a high Ki-67 labeling index (≥38%) is significantly associated with lower recurrence-free and disease-specific survival rates, making it an independent predictor of recurrence risk [47]. Additionally, a ratio combining Ki-67 with other immunohistochemical markers—ER, PR, and p53—was found to effectively predict lymph node metastasis in early-stage cases, with an AUC as high as 0.876 [48]. Another study reported that high Ki-67 expression correlates with higher tumor grade and ER-negative status, although no clear association with overall prognosis was observed [49]. In benign conditions, Ki-67 expression in endometrial polyps has been linked to clinical symptoms such as abnormal bleeding, suggesting its role in indicating tissue proliferative activity [50]. Collectively, these studies underscore the clinical utility of Ki-67 in prognosis, treatment decision-making, and pathological evaluation of EC and related conditions.

CD31, also known as platelet endothelial cell adhesion molecule-1, is a well-established immunohistochemical marker for vascular endothelial cells and is widely used to assess microvessel density (MVD) as an indicator of tumor angiogenesis. In EC, higher CD31 expression has been associated with increased tumor aggressiveness, including deeper myometrial invasion, higher histological grade, and lymph node metastasis [51]. Elevated CD31 expression reflects enhanced nervousness, which promotes tumor growth and facilitates dissemination. Recent studies have also demonstrated that increased CD31-positive MVD is significantly correlated with worse clinical outcomes in endometrioid EC, highlighting its role as a potential prognostic marker [46]. Collectively, these findings support the utility of CD31 as both an angiogenic indicator and a predictor of tumor invasiveness and adverse prognosis in EC.

Fibronectin, a major extracellular matrix glycoprotein, plays a crucial role in the progression of EC by promoting tumor invasion, EMT, and stromal remodeling. One study demonstrated that fibronectin secreted by SPARC-expressing endometrial cancer cells activates stromal fibroblasts and enhances EMT, thereby facilitating cancer cell migration and metastasis [6]. Clinically, elevated serum fibronectin levels have been observed in patients with malignant endometrial lesions compared to those with benign conditions, suggesting its potential utility as a diagnostic and prognostic biomarker [52]. Additionally, fibronectin’s interaction with integrins such as α5β1 contributes to angiogenesis and tumor progression through the activation of downstream signaling pathways [53]. These findings indicate that fibronectin plays a crucial role in the invasive capacity of EC by enhancing the tumor microenvironment and stimulating cellular mechanisms involved in tissue infiltration.

In our study, patients with high HOXA5 expression exhibited better survival outcomes, which contrasts with the previously reported association between high Ki-67 expression and poor prognosis in endometrial cancer. We discuss this discrepancy in the sections that follow.

Although high HOXA5 expression in our study was significantly associated with increased Ki-67 levels (*p* = 0.001)—a marker typically indicative of enhanced cellular proliferation—it was paradoxically linked to better overall survival outcomes (*p* = 0.026). This finding contrasts with previous studies suggesting that elevated Ki-67 expression is associated with poor prognosis in EC [47]. A plausible explanation lies in the broader regulation of the tumor microenvironment mediated by HOXA5. Specifically, tumors with high HOXA5 expression exhibited significantly lower levels of CD31 (*p* = 0.007) and fibronectin (*p* = 0.001), which are markers associated with angiogenesis and extracellular matrix remodeling, respectively. These findings suggest that while HOXA5 may enhance tumor cell proliferation, it may also suppress the angiogenic and invasive potential of tumors by modulating stromal components, ultimately limiting tumor progression and metastasis.

However, it is noteworthy that neither CD31 nor fibronectin emerged as independent prognostic factors in multivariable logistic regression analysis. This discrepancy may be attributed to the multifactorial nature of HOXA5’s biological activity, which likely exerts its prognostic impact through the coordinated modulation of multiple signaling pathways, rather than through isolated effects on individual markers. Moreover, the expression of CD31 and fibronectin may also be influenced by additional tumor characteristics, such as histologic grade or molecular subtype, which could attenuate their statistical significance in multivariable models. Collectively, these results support a dual role for HOXA5 in promoting proliferation while restraining the tumor’s invasive and angiogenic capacity, potentially explaining the improved survival observed despite increased Ki-67 expression.

This study has several limitations that should be acknowledged. First, the relatively small sample size (n = 75) may limit the generalizability of our findings and reduce the statistical power to detect subtle associations, particularly in multivariable analyses. While we agree that sample size is a major constraint, we would like to highlight that our tissue microarrays were custom constructed in-house, with each tissue core measuring 2 mm in diameter—larger than those found in most commercial arrays—to reduce sampling errors and minimize tissue heterogeneity. Second, although our immunohistochemical results suggest that high HOXA5 expression is associated with reduced CD31 (*p* = 0.007) and fibronectin (*p* = 0.001) levels—markers potentially linked to decreased angiogenesis and extracellular matrix remodeling—these associations did not remain statistically significant in multivariable logistic regression analysis (CD31: *p* = 0.063; fibronectin: *p* = 0.057), leaving the independent prognostic value of these markers inconclusive. Finally, we did not conduct mechanistic in vitro or in vivo studies to directly verify the functional role of HOXA5 in modulating tumor biology. Further basic research is needed to clarify the molecular mechanisms underlying these observations.

## 5. Conclusions

Our findings suggest that HOXA5 plays a complex and context-dependent role in endometrial cancer (EC). While high HOXA5 expression was independently associated with elevated Ki-67 levels—typically indicative of increased proliferative activity—it was paradoxically correlated with improved overall survival. This seemingly contradictory outcome may be explained by HOXA5’s broader tumor-suppressive functions. As supported by prior studies, HOXA5 enhances TP53 expression and activity, modulates hormonal signaling via progesterone receptors, and suppresses key oncogenic pathways including ERBB2 and L1CAM. In our cohort, high HOXA5 expression was also linked to significantly lower levels of CD31 and fibronectin, suggesting impaired angiogenic and stromal support for tumor invasion. Although CD31 and fibronectin did not retain independent prognostic value in multivariable analysis, their downregulation in the high-HOXA5 group may reflect a biologically less aggressive tumor phenotype. Collectively, these findings support a dual modulatory role of HOXA5 in EC—enhancing proliferation under regulated conditions, while concurrently limiting the vascular, stromal, and metastatic infrastructure required for aggressive progression. HOXA5 may therefore serve not only as a prognostic biomarker but also as a candidate for targeted therapeutic development in EC.

## Figures and Tables

**Table 1:** Primary antibodies used for immunohistochemical analysis in endometrial cancer tissue samples.

Antibody	Manufacturer	Country	City	Catalog Number	Dilution
HOXA5	Abcam	UK	Cambridge	ab82645	1:50
Caspase-3	Cell Signaling	USA	Danvers, MA	9664	1:100
Ki67	BioLegend	USA	San Diego, CA	350503	1:100
CD31	Abbiotec	USA	San Diego, CA	250590	1:500
E-cadherin	Abcam	UK	Cambridge	ab40772	1:100
N-cadherin	Abcam	UK	Cambridge	ab76011	1:100
Fibronectin	Santa Cruz	USA	Dallas, TX	SC-8422	1:50
pAkt	GeneTex	USA	Irvine, CA	GTX11901	1:50
pErk	R&D	USA	Minneapolis, MN	AF1018	1:200
pStat3	Abcam	UK	Cambridge	ab76315	1:50
pAMPK	Cell Signaling	USA	Danvers, MA	2535	1:100

**Table 2:** Comparison of clinicopathological characteristics between low- and high-HOXA5 expression groups in endometrial cancer patients.

	Low Expression of HOXA5 (N = 30)	High Expression of HOXA5 (N = 45)	Total (N = 75)	*p*-Value
Age	56.5 (50.5, 64.0)	56.0 (50.0, 59.5)	56.0 (50, 60)	0.482
Grading				0.631
Well	6 (20.0%)	12 (26.7%)	18 (24.0%)	
Moderate	17 (56.7%)	26 (57.8%)	43 (57.3%)	
Low	7 (23.3%)	7 (15.6%)	14 (18.7%)	
Size	3.8 (1.4, 6.6)	2.8 (1.6, 5.0)	3.0 (1.5, 5.0)	0.661
Stage				0.796
I	24 (80.0%)	37 (82.2%)	61 (81.3%)	
II	2 (6.7%)	4 (8.9%)	6 (8.0%)	
III	4 (13.3%)	4 (8.9%)	8 (10.7%)	

**Table 3:** Comparison of tumor biomarker expression between low- and high-HOXA5 expression groups in endometrial cancer.

	Low Expression of HOXA5	High Expression of HOXA5	Total	*p*-Value
Caspase-3	6.3 (4.5, 9.3)	8.6 (5.4, 13.7)	7.1 (5.2, 12.8)	0.062
Ki67	2.3 (0.3, 7.1)	9.0 (1.7, 24.4)	5.2 (1.3, 16.1)	0.001 **
CD31	8.2 (6.4, 14.8)	6.4 (4.8, 8.4)	6.9(5.1, 96)	0.007 **
E-cad	108.5 (101.6, 115.6)	110 (105.8, 118.5)	109.4 (103.5, 116.1)	0.216
N-cad	8.9 (3.5, 19.9)	7.3 (2.5, 40.6)	7.6 (2.5, 34.1)	0.665
Fibronectin	16.6 (6.3, 51.5)	2.9 (1.1, 23.4)	8.9 (2.1, 31.9)	0.001 **
pAkt	2.5 (1.2, 3.6)	4.1 (1.9, 6.8)	3.0 (1.6, 6.5)	0.031 *
pErk	0.2 (0.0, 2.8)	0.7 (0.2, 5.3)	0.4 (0.1, 4.6)	0.069
pStat3	0.2 (0.1, 0.4)	0.1 (0.1, 0.3)	0.1 (0.1, 0.4)	0.414
pAMPK	16.1 (13.2, 25.4)	13.6 (10.0, 21.9)	14.8 (12.0, 22.6)	0.218

* *p* < 0.05, ** *p* < 0.01.

**Table 4:** Univariable and multivariable logistic regression analysis of factors associated with high HOXA5 expression in endometrial cancer.

	Univariable	*p*-Value	Multivariable	*p*-Value
Age	0.98 (0.93–1.03)	0.329		
Grading				
Well	Reference			
Moderate	0.77 (0.24–2.43)	0.649		
Low	0.50 (0.12–2.10)	0.344		
Size	0.94 (0.80–1.11)	0.450		
Stage				
I	Reference			
II	1.30 (0.22–7.64)	0.774		
III	0.65 (0.15–2.84)	0.566		
Marker				
Caspase-3	1.07 (0.99–1.16)	0.088		
Ki67	1.08 (1.02–1.14)	0.008 **	1.12 (1.03–1.19)	0.004 **
CD31	0.91 (0.83–0.98)	0.018 *	0.89 (0.78–1.01)	0.063
E-cad	1.04 (0.98–1.11)	0.189		
N-cad	1.01 (0.99–1.03)	0.241		
Fibronectin	0.98 (0.97–1.00)	0.021 *	0.98 (0.96–1.00)	0.057
pAkt	1.02 (0.95–1.09)	0.597		
pErk	1.01 (0.97–1.05)	0.628		
pStat3	0.75 (0.50–1.13)	0.163		
pAMPK	0.97 (0.92–1.02)	0.257		

* *p* < 0.05, ** *p* < 0.01.

## Data Availability

The original contributions presented in this study are included in the article/Supplementary Materials. Further inquiries can be directed to the corresponding authors.

## Supplementary Materials

The following supporting information can be downloaded at https://www.mdpi.com/article/10.3390/cancers17152473/s1: Table S1: Effect size analysis (Cohen’s d and Cramér’s V) between HOXA5 high and low expression groups for clinicopathological and molecular variables.

## Abbreviations

The following abbreviations are used in this manuscript:

EC	Endometrial cancer
MVD	Microvessel density
EMT	Epithelial–mesenchymal transition
