# Supplementary material for: HOXA5 as a Dual Modulator of Tumor Biology in Endometrial Cancer
Source: Cancers (Basel). 2025 Jul 26;17(15):2473. doi: 10.3390/cancers17152473 (PMC12346555; doi:10.3390/cancers17152473)
Supplement: Supplementary file 1 [file cancers-17-02473-s001.zip › cancers-3719091-supplementary.pdf]

Supplementary materials

Table S1. Effect size analysis (Cohen's d and Cramér's V) between HOXA5 high and low expression groups for clinicopathological and molecular variables.

**Variable**	**Cohen's d**
Age	−0.23029
Size	−0.17692
caspase3	0.42182012396627344
Ki67	0.7101887999314216
CD31	−0.63779
E-cad	0.3126328358691567
N-cad	0.2786979787621497
Fibronectin	−0.60082
pAkt	0.12390277701977867
pErk	0.11333070121165702
pStat3	−0.4041
pAMPK	−0.26836
Variable	Cramér's V
Stage	0.13
